# Iris metastasis of diffuse large B-cell lymphoma misdiagnosed as primary angle-closure glaucoma: A case report and review of the literature

**DOI:** 10.1515/biol-2021-0008

**Published:** 2021-01-20

**Authors:** Zebing Li, Zhongjing Lin, Yisheng Zhong, Xi Shen

**Affiliations:** Department of Ophthalmology, Ruijin Hospital, Affiliated Shanghai Jiaotong University School of Medicine, 197 Ruijin Er Road, 200025, Shanghai, China

**Keywords:** lymphoma, intraocular iris metastasis, glaucoma

## Abstract

**Background:**

Lymphoma with intraocular metastasis is an uncommon and serious disease. We describe a case of diffuse large B-cell lymphoma (DLBCL) with iris metastasis. Meanwhile, we refer to published case reports retrieved via a PubMed search to summarize this rare disease.

**Case presentation:**

Glaucoma and uveitis symptoms were found in the left eye of a 50-year-old woman upon admission to the hospital. After treatment and pathological examination, the iris of her left eye was diagnosed with DLBCL. Given the patient’s unfavorable treatment options in the local hospital, primary enucleation was offered as a therapeutic option.

**Conclusions:**

Iris metastasis of systemic lymphoma is an extremely rare ophthalmic disease with poor prognosis. Ophthalmologists should be able to definitively and differentially diagnose eye symptoms and pay attention to systemic conditions to provide a series of optimized treatments.

## Abbreviations

DLBCL : diffuse large B-cell lymphoma
NHL : non-Hodgkin lymphoma
PET-CT : positron-emission tomography-computer tomography
PACG : primary angle-closure glaucoma
IOP : intraocular pressure
Nd:YAG : neodymium-doped yttrium aluminum garnet
IOL : intraocular lens
UBM : ultrasound biomicroscopy
HE : hematoxylin–eosin
UGH : uveitis–glaucoma–hyphema
MCL : mantle cell lymphoma
NK : natural killer

## 1 Background

Non-Hodgkin lymphoma (NHL) refers to a group of cancers that derive from lymphocytes and often localize to lymph nodes. NHL accounts for approximately 5.1% of all cancer cases and 2.7% of all cancer deaths [1]. NHL that involves the eyes commonly affects the uvea and vitreous body. Histopathologically proven ocular involvement has been found in 7% of patients with systemic lymphoma at the time of death [2]. Uveal lymphoma is confined to the choroid in most cases, and reports of iris involvement associated with this condition are limited to isolated cases or small case series [3]. There is a consensus that iris lymphoma can be divided into primary and secondary types. After consulting the literature, we found that few cases involving iris metastasis of systemic lymphoma have been reported.

Therefore, we are able to present this interesting case of iris metastasis of systemic lymphoma in a Chinese woman. We focus on this patient’s clinical symptoms and therapeutic process to strengthen our understanding of lymphoma involving the iris and discuss advanced treatment options.

## 2 Case presentation

A case report of iris metastasis of systemic lymphoma in a Chinese woman is presented. The patient had a 6-month history of diffuse large B-cell lymphoma (DLBCL), and a positron emission tomography-computer tomography (PET-CT) scan and bone marrow biopsy after six cycles of chemotherapy were negative for recurrence.

**Figure 1:**
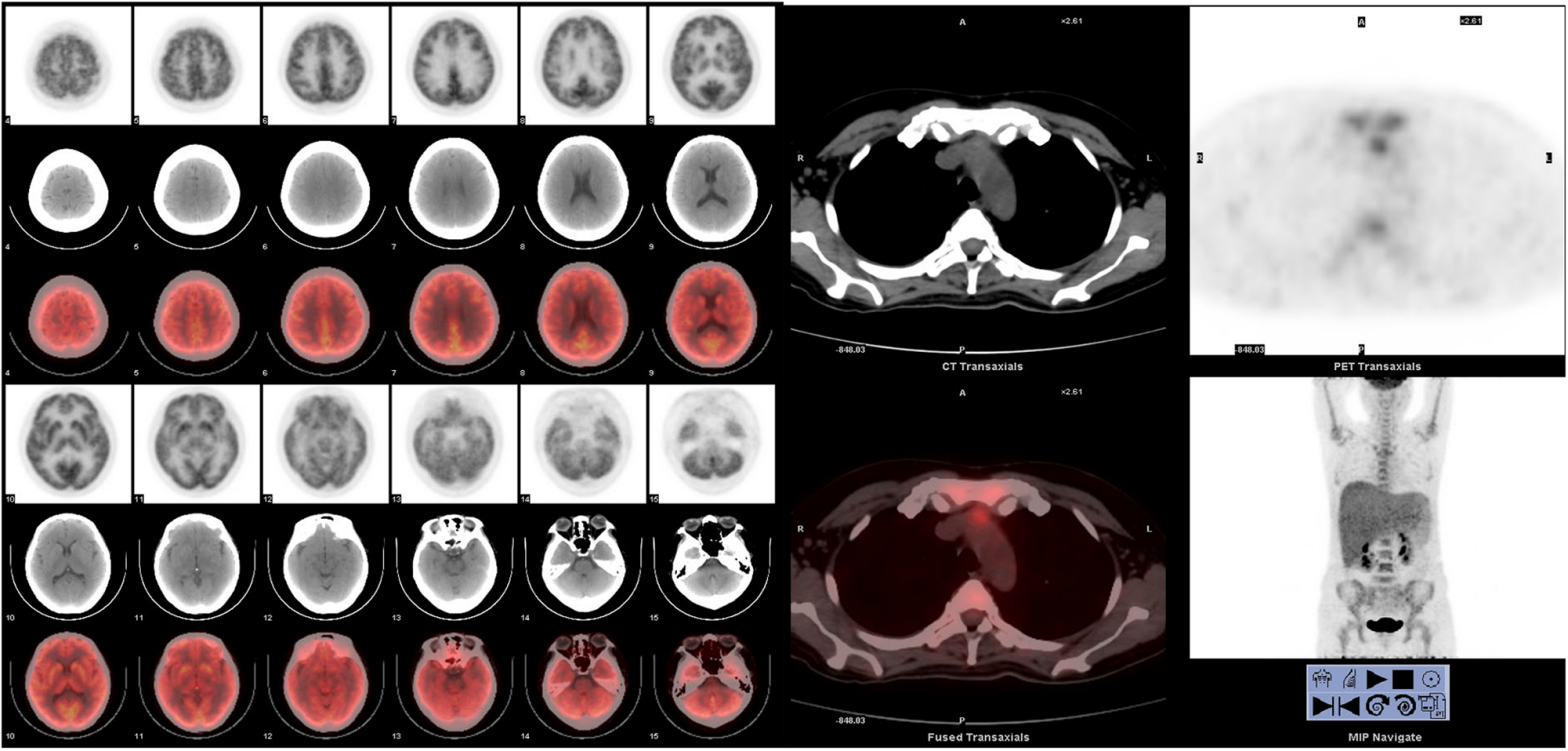
The patient’s first PET-CT report indicated that the systemic primary lesion was found before the mediastinum, and no abnormal hypermetabolism was found in the brain.

**Figure 2:**
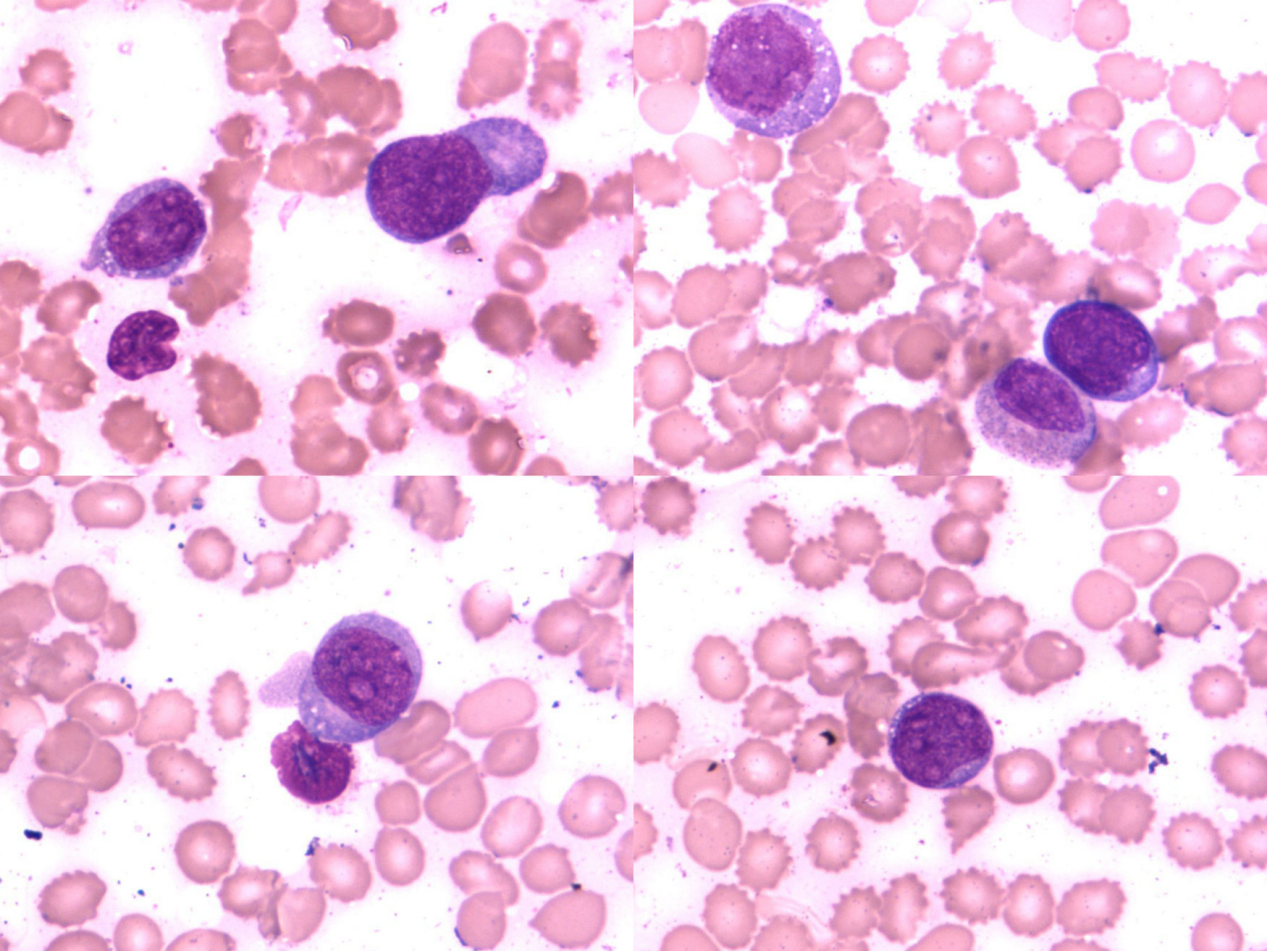
The flow cytology showed typical lymphoma cells.

It is worth noting that the systemic primary lesion was found in the anterior mediastinum from the PET-CT image (Figure 1). The flow cytometry analysis indicated that CD19-positive cells accounted for about 4.6% of the nucleated cells. All the lymphocytes expressed HLA-DR, CD5, CD19, CD20, CD22, sLamda, and cCD79a; some cells expressed CD38; but all cells were negative for CD23, FCM7, BCL-2, CD103, and CD200. These lymphocytes were considered to be abnormal monoclonal B lymphocytes. Bone marrow biopsy histopathologic examination of the tissue revealed partial positive staining for MPO and MUM; positive staining for CD19, CD20, and CD79α; 50% positive staining for Ki-67; positive staining in a small number of cells for CD3, CD7, and Pax-5; and negative staining for CD34, S-100, CD56, HCK, LCK, BCL-6, BCL-2, CD22, CD23, CD5, CyclinD1, c-myc, and CD99. Combining the flow cytometry with the bone marrow biopsy histopathologic examination, the diagnosis was B-cell lymphoma (tendency toward DLBCL) with involvement of the bone marrow (Figure 2).

Our patient, a 50-year-old woman, accepted PET-CT again to evaluate the efficacy of chemotherapy in November 2016, and the result was negative. During the preceding 20 days in February 2017, she presented to a local hospital with a chief complaint of pain and redness in her left eye. She was diagnosed with primary angle-closure glaucoma (PACG) and acute iridocyclitis. After the patient was treated with intraocular pressure (IOP)-lowering drugs (including timolol maleate, brimonidine tartrate, and brinzolamide eye drops) and neodymium-doped yttrium aluminum garnet (Nd:YAG) laser iridectomy, her IOP remained uncontrolled. She complained of much more pain and headache. To control her IOP and diminish pain, a local ophthalmologist performed phacoemulsification and intraocular lens (IOL) implantation combined with trabeculectomy in local anesthesia. Adjunctive 5-fluorouracil was administered intraoperatively. However, the curative effects of these procedures were temporary and she still felt unbearable pain in her left eye; therefore, the patient visited our hospital after 1 week.

**Figure 3:**
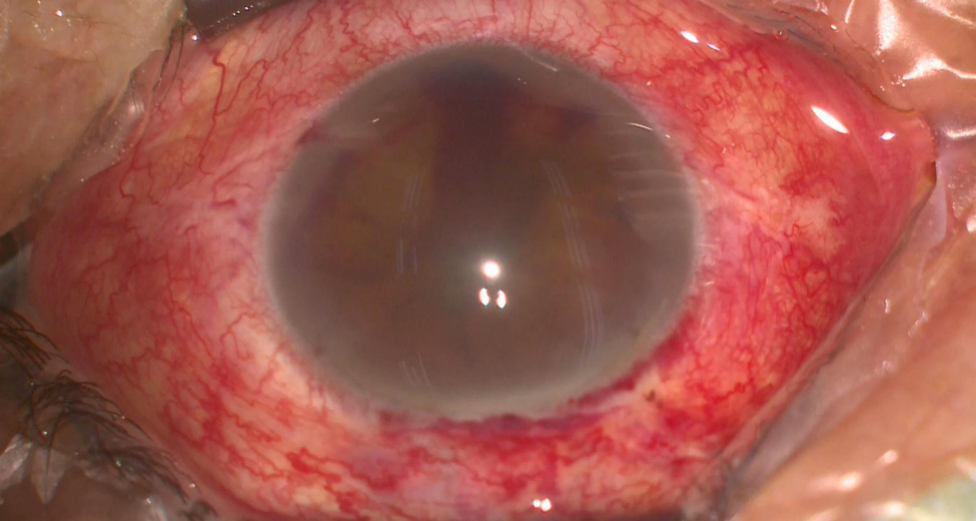
Photograph of the left eye at presentation; anterior segment examination of the left eye revealed conjunctival hyperemia, corneal edema, and hyphema.

**Figure 4:**
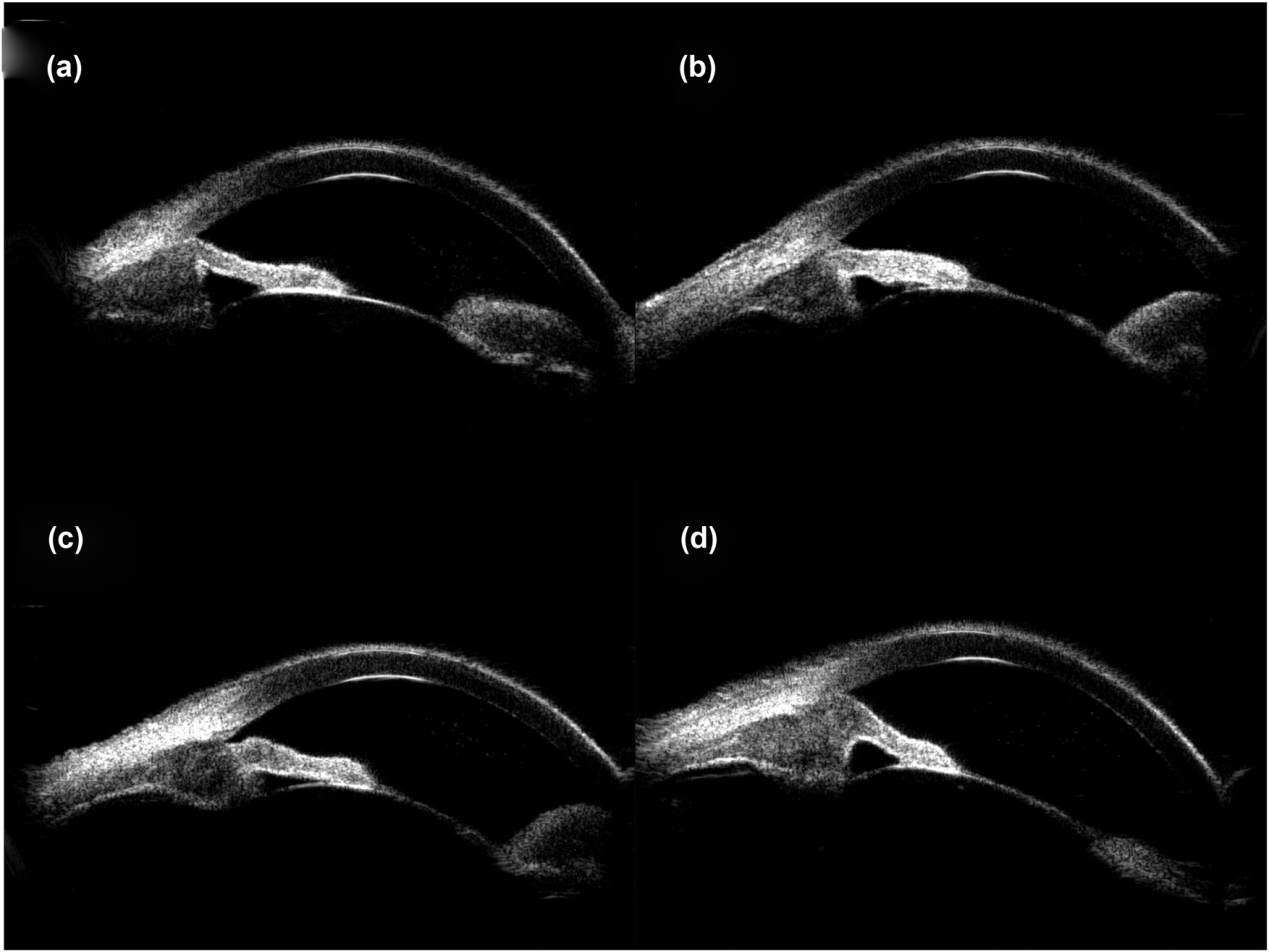
UBM images obtained clockwise from 12:00 showed apophysis of the roots of the ciliary body of the right eye and closure of the anterior chamber angle. (a) 12:00, (b) 3:00, (c) 6:00, and (d) 9:00.

**Figure 5:**
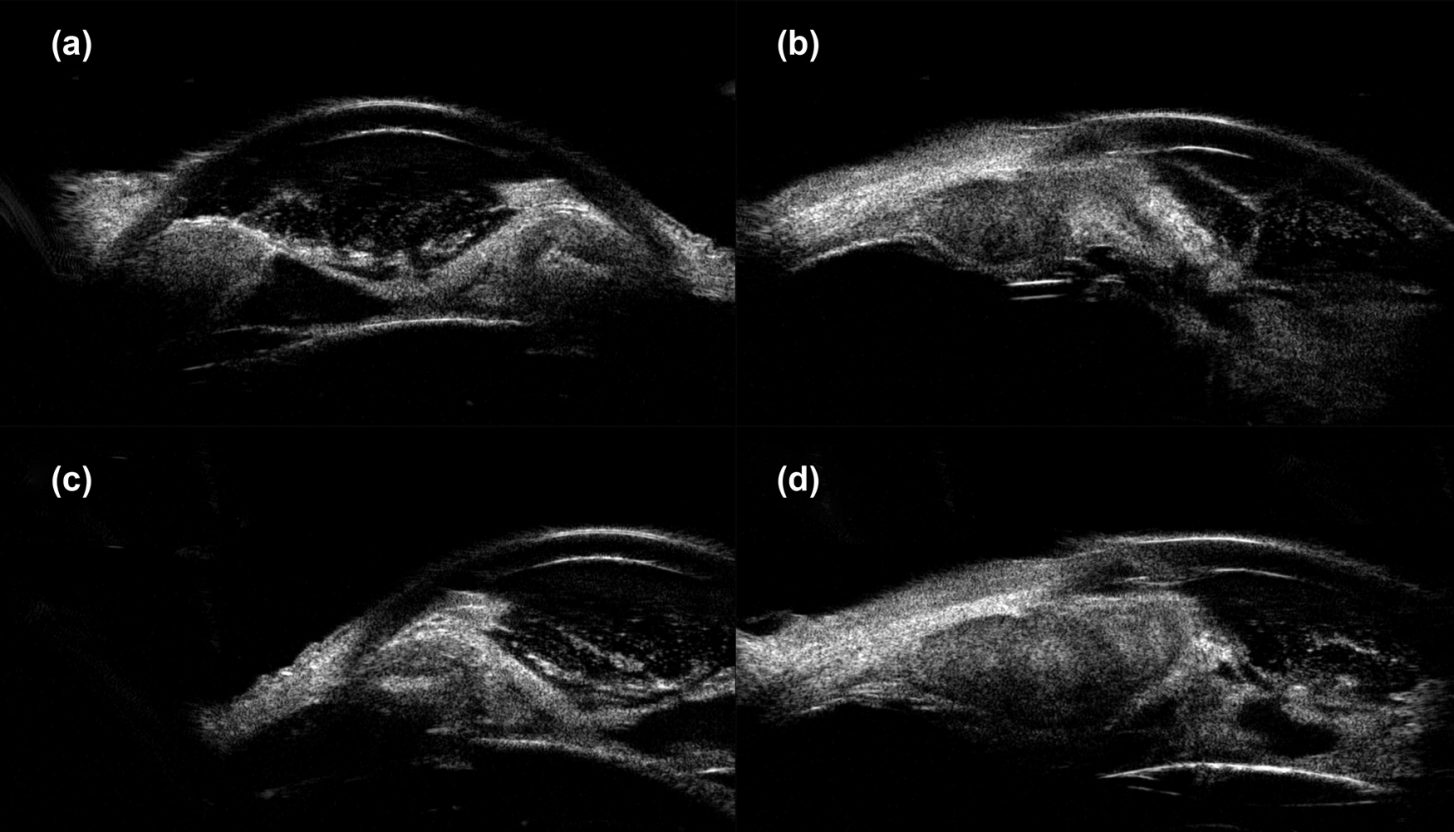
Images obtained clockwise from 12:00 showed apophysis of the roots of the ciliary body of the left eye, anatomical disorder, and closure of the anterior chamber angle. (a) 12:00, (b) 3:00, (c) 6:00, and (d) 9:00.

The patient’s unaided vision acuity was 20/20 in the right eye, with light perception in the left eye. IOP was 11.3 mm Hg in the right eye and 50.5 mm Hg in the left eye. Anterior segment examination of the left eye revealed conjunctival hyperemia, corneal edema, and hyphema (Figure 3). High-frequency (50 MHz) ultrasound biomicroscopy (UBM) indicated that the roots of the ciliary body were intumescent in both the eyes, and the anterior chamber angles were closed in all orientations. The anatomical structure of the left eye was completely destroyed (Figures 4 and 5).

**Figure 6:**
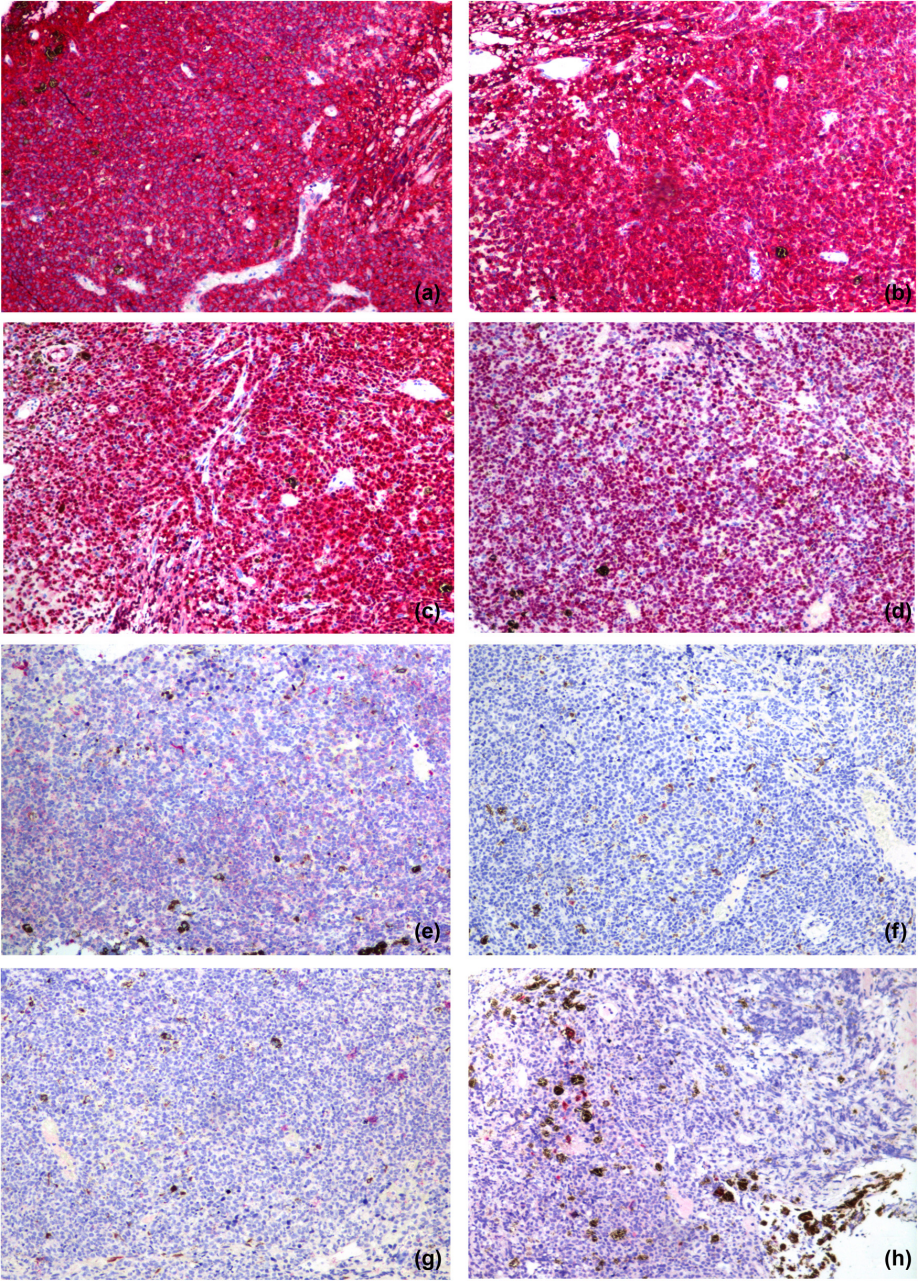
Histopathologic examinations of the tissue showed positive staining for CD20 (×100) (a) and CD79a (×100) (b); and >90% staining for MUM-1 (×100) (c) and BCL-6 (×100) (d); and negative staining for CD10 (×100) (e). There was negative staining for the melanoma-specific immunity indicators HMB45 (×100) (f), MelanA (×100) (g), and S100 (×100) (h).

**Figure 7:**
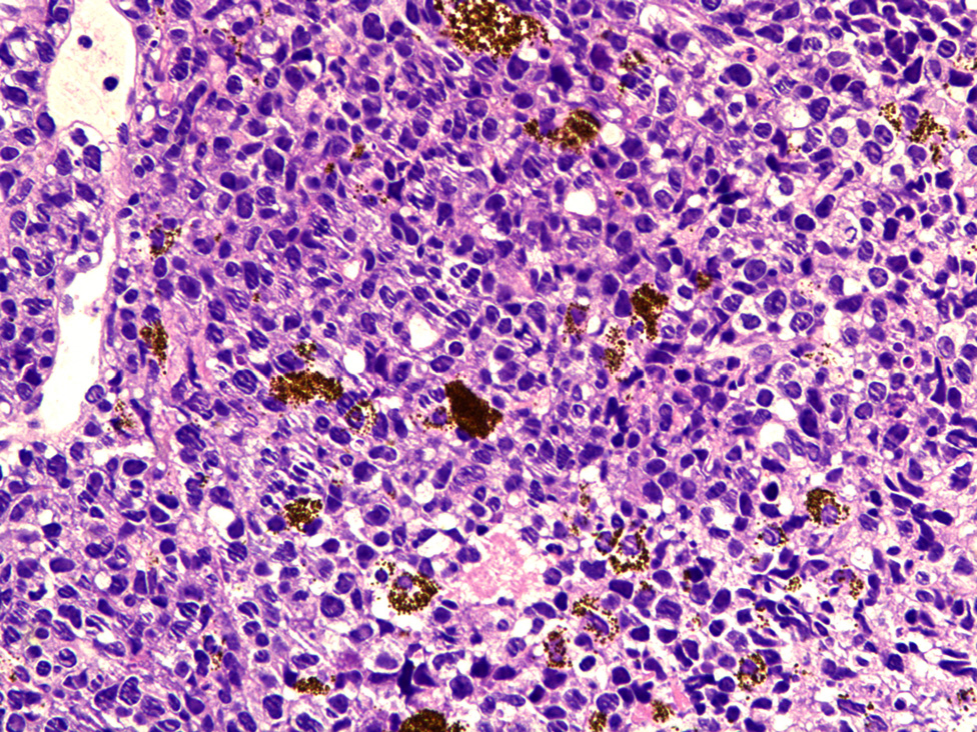
HE staining showed massive malignant lymphoma cells (×400).

Rare involvement of a systemic disease was strongly suspected based on the patient’s history of lymphoma, her recurrent glaucoma symptoms, and the roots of the ciliary body of the left eye. At the time, differential diagnosis included secondary lymphomatous involvement of the iris and amelanotic melanoma. To clearly establish a diagnosis, a tissue biopsy of the iris was attempted in the operating room but was unsuccessful due to hard tissue density. As a result, intraocular scissors were used to obtain an incisional tissue biopsy instead. Histopathologic examination of the tissue showed positive staining for CD20 (×100; Figure 6a) and CD79a (×100; Figure 6b); and >90% staining for MUM-1 (×100; Figure 6c) and BCL-6 (×100; Figure 6d); but negative staining for CD10 (×100; Figure 6e). Negative staining was also observed for the melanoma-specific immunity indicators HMB45 (×100; Figure 6f), MelanA (×100; Figure 6g), and S100 (×100; Figure 6h). Hematoxylin–eosin (HE) staining showed massive malignant lymphoma cells (×400; Figure 7). These results led to a diagnosis of DLBCL.

Based on the tissue biopsy report, the final treatment option was up to the patient and her family to decide. After explaining the possible advantages and outcomes of different treatment options for the management of the disease, we suspected the recurrence of lymphoma and suggested another PET-CT. However, the patient’s family refused our suggestion because of the poor prognosis (including the worsening of eye condition and the complications of systemic lymphoma [3,4,5,6,7,8]) and the high cost (after six cycles of chemotherapy, her family was deep in debt). The patient finally accepted MRI examination of the eyes and the brain in time to ensure the tumor had not spread to the brain or into the head. The result was negative. We also suggested anti-VEGF agents to inhibit neovascularization, but the patient rejected anti-VEGF agents and systemic chemotherapy for the same reason. Based on the reported cases in the literature search, there is no consensus about an optimal therapeutic approach. An ophthalmologist suggested radiotherapy for the patient to stabilize her symptoms consisting of about 40 Gy external beam irradiation delivered evenly in 20 fractions over a period of 1 month. Due to the uncertainty about the efficacy and side effects of radiation therapy, our patient did not accept the regimen. Eventually, given the poor visual potential of the left eye and the presence of neovascular glaucoma, primary enucleation was offered as a therapeutic option. The patient accepted this option. To facilitate follow-up care, the patient returned to the local hospital after 2 weeks for enucleation. She died in July 2018. Before her death, the patient never came back to our institute, and this information was made available to us only when we contacted the family of the patient. Therefore, information on further management and follow-up could not be obtained.


**Informed consent:** Informed consent has been obtained from all individuals included in this study.

## 3 Discussion

In a search of the PubMed database, we found 52 reports of lymphoma associated with the iris, including 12 reports of primary B-cell intraocular lymphoma (23.1%), 12 reports of primary T-cell intraocular lymphoma (23.1%), and 6 reports of lymphoma with intraocular metastasis (11.5%). We found 7 reports addressing the method for diagnosing intraocular lymphoma (13.4%), 1 report of primary intraocular lymphoma with concurrent involvement of both B-cells and T-cells (1.9%), 3 reports of unspecified pathological patterns (5.8%), and 11 reports of other pathological patterns (21.2%), such as concurrent mucosa-associated lymphoid tissue lymphoma, melanoma, and plasmacytoma involving the uvea. It was evident that most of these reported cases involved primary intraocular lymphoma; only six cases found via the PubMed search involved patients diagnosed with lymphoma with intraocular metastasis [9,10,11,12,13,14]. Thus, it is important to discuss this patient, analyze her clinical manifestations, study treatment procedures, and learn from our experience.

Gauthier et al. [9] reported a patient with recurrent hyphema and uncontrolled IOP who was eventually found to have a rapidly growing iris mass. The patient appeared to have uveitis–glaucoma–hyphema (UGH) syndrome, and this masqueraded as UGH syndrome, which could mislead an ophthalmologist with respect to diagnosis and treatment. Agarwal et al. [10] reported a patient who presented with an aggressive, relapsing, metastatic, systemic, blastic variant of mantle cell lymphoma (MCL) with ocular involvement; large tumor cells were present in the anterior chamber, and hypopyon and fibrin were also observed. These authors suggested that combination therapy with intravitreal injections of chemotherapeutic agents (methotrexate and rituximab) targeting the monoclonal B-cell population and novel systemic agents (ibrutinib) may help to achieve remission in cases involving anterior segment metastasis of aggressive subtypes of NHL. Matsui et al. [11] reported a similar case in which a patient visited an ophthalmology clinic with the chief complaint of blurred vision in the left eye which was affected by neovascular glaucoma. This patient had a history of systemic malignant lymphoma. UBM revealed abnormal thickening of the iris, and cytological examination of the aqueous humor revealed invasion by malignant cells that were presumed to be of lymphocyte origin. Radiation therapy was administered to the left eye, and IOP was normalized, with reductions in neovascularization of the iris and thickening of the ciliary body observed within a week. Nakanishi et al. [12] reported a patient who had previously been diagnosed with nasal lymphoma. The patient developed resistance to steroid therapy and nodular masses in the left iris. UBM and an iris biopsy were performed, leading to a diagnosis of natural killer (NK)-cell lymphoma. Clinical ophthalmologists should consider metastatic malignancy in differential diagnosis when a patient has developed resistance to steroid therapy. Bawankar et al. [13] reported a case of spontaneous hyphema associated with anterior uveitis presenting in a 69-year-old female as the prominent sign of the intraocular spread of systemic DLBCL. She had a history of diabetes and was initially misdiagnosed with neovascular glaucoma. Pei et al. [14] reported a 59-year-old Han Chinese male with past history of systemic MCL with complaints of redness, pain, and blurred vision in the left eye. UBM and B-scan were then performed and ciliary body masses in both eyes were detected. Combined liquid-based cytology tests and gene rearrangement assays of the aqueous humor specimen confirmed this to be a B-cell malignancy. After radiation and chemotherapy, the disease was in remission. The six aforementioned reports not only indicate that lymphoma with intraocular metastasis is a rare disease but also provide specific approaches regarding the diagnosis and treatment of this condition.

Cooper and Ricker were the pioneers who first reported intraocular lymphoma in 1951, and Roppart et al. named this condition reticulum cell sarcoma. Currently, it has been confirmed that most cases of intraocular lymphoma are attributable to B lymphocytes, with few cases involving T-cell or U-cell lymphoma [15]. Given the aforementioned studies, we can conclude that for lymphoma that involves the iris, the typical presentation includes a bulging mass in certain quadrants of the iris with accompanying clinical manifestations such as diminution of vision, anterior uveitis, and neovascular glaucoma [11,16,17,18]. The gold standard for diagnosis is biopsy, including fine-needle aspiration biopsy, tissue biopsy, and other types of biopsies [16,17,18]. Based on the reported cases, the optimal therapeutic approach is still being explored. Radiotherapy has been reported to have an outstanding curative effect because of the radiosensitivity of lymphoma [12,18,19,20].

In our case, we emphasize the diagnosis and treatment process. The patient had a history of DLBCL, iris bulging, and NHL involving the anterior chamber of the iris that masqueraded as PACG. This misdiagnosis affected the treatment plan. The patient accepted Nd:YAG laser iridectomy; however, the laser hole in the iris was rapidly blocked by effusion. It was strongly suspected that this effusion could be attributable to monoclonal lymphocyte hyperplasia and infiltration due to stimulation from the YAG surgery and the powerful proliferative potential of these cells; as a result, the filtration pathway may get blocked shortly after the Nd:YAG laser iridectomy. Phacoemulsification cataract surgery combined with trabeculectomy undoubtedly aggravated lymphocyte diffusion, resulting in a poor prognosis. The optimal opportunity to perform radiotherapy may have been missed. On the other hand, as we know, most malignant tumors are rich in blood supply. Both neovascular glaucoma and secondary angle-closure glaucoma may be involved. Anti-VEGF agents have shown some benefit in the management of neovascular glaucoma but still cannot solve the fundamental problem of the tumor tissue. In addition, anti-VEGF agents need to be administered many times, and continuing costs are particularly problematic. The effect of anti-VEGF agents needs to be further explored for this type of disease.

During the process of cachexia, a consecutive series of complications could develop in patients such as cataract formation, maculopathy, vitreous hemorrhage, neovascular glaucoma, and sterile endophthalmitis, all of which could cause serious loss of vision or even blindness [4,5,6]. The iris neoplasm associated with systemic lymphoma tends to be very aggressive, not only leading to ocular complications but also resulting in the complications of systemic lymphoma [3,7]. The prognosis is poor, and most patients suffer from recurrence, metastasis, and death. Median survival time for this condition is 12–24 months [8]. Thus, primary enucleation is a common and relatively safe therapeutic option.

The biopsy of tissue from our patient indicated that tumor cells were distributed such that they permeated much of the iris pigment; these cells were not of uniform size and had anomalous nuclei. For diagnosis, the patient’s condition needed to be differentiated from melanoma. Immunohistochemistry revealed negative staining for the melanoma-specific markers HMB, MelanA, and S100. Positive staining for CD20 and CD79a indicated that the tumor was derived from B lymphocytes. Negative staining was observed for CD10, whereas over 90% staining was detected for MUM-1 and BCL-6. Therefore, the pathological diagnosis was DLBCL.

## 4 Conclusions

This case demonstrated that lymphomatous involvement of the iris is a rare phenomenon that can be difficult to distinguish from more common causes of anterior uveitis and neovascular glaucoma. Despite its rarity, iris metastasis of systemic lymphoma should be considered in the differential diagnosis of an iris mass. Our report intends to demonstrate the experience and lessons gained in the diagnosis and treatment process. We summarize this rare disease with the help of a literature review and hope to provide more informational support for clinical ophthalmologists. Interdisciplinary differential diagnosis is a key element for immediate therapy to improve the mostly poor prognosis of this disease.
